# Oral paracetamol and/or ibuprofen for treating pain after soft tissue injuries: Single centre double-blind, randomised controlled clinical trial

**DOI:** 10.1371/journal.pone.0192043

**Published:** 2018-02-06

**Authors:** Kevin K. C. Hung, Colin A. Graham, Ronson S. L. Lo, Yuk Ki Leung, Ling Yan Leung, S. Y. Man, W. K. Woo, Giles N. Cattermole, Timothy H. Rainer

**Affiliations:** Accident and Emergency Medicine Academic Unit, Chinese University of Hong Kong, Shatin, New Territories, Hong Kong SAR; Dartmouth-Hitchcock Medical Center, UNITED STATES

## Abstract

**Background:**

Soft tissue injuries commonly present to the emergency department (ED), often with acute pain. They cause significant suffering and morbidity if not adequately treated. Paracetamol and ibuprofen are commonly used analgesics, but it remains unknown if either one or the combination of both is superior for pain control.

**Objectives:**

To investigate the analgesic effect of paracetamol, ibuprofen and the combination of both in the treatment of soft tissue injury in an ED, and the side effect profile of these drugs.

**Methods:**

Double-blind, double dummy, placebo-controlled randomised controlled trial. 782 adult patients presenting with soft tissue injury without obvious fractures attending the ED of a university hospital in the New Territories of Hong Kong were recruited. Patients were randomised using a random number table into three parallel arms of paracetamol only, ibuprofen only and a combination of paracetamol and ibuprofen in a 1:1:1 ratio. The primary outcome measure was pain score at rest and on activity in the first 2 hours and first 3 days. Data was analysed on an intention to treat basis.

**Results:**

There was no statistically significant difference in pain score in the initial two hours between the three groups, and no clinically significant difference in pain score in the first three days.

**Conclusion:**

There was no difference in analgesic effects or side effects observed using oral paracetamol, ibuprofen or a combination of both in patients with mild to moderate pain after soft tissue injuries attending the ED.

**Trial registration:**

The study is registered with ClinicalTrials.gov (no. NCT00528658).

## Introduction

Soft tissue injuries comprises of various types of injuries including contusions, sprains, crush injuries, cuts and lacerations. They can occur in different body regions including the limbs, neck and back, or the trunk. Specific guidelines and recommendations are available for the treatment for low back pain [1–3], ankle and foot injuries [4, 5] etc, but oral analgesics are often recommended for the relief of acute pain. In a previous review, Ducharme highlighted the problem of under-treatment of pain in the emergency department (ED), and provided suggestions for pain relief and analgesic use [6]. Non-steroidal anti-inflammatory drugs (NSAID) and paracetamol are commonly used analgesic drugs in the ED [7].

NSAIDs are cyclo-oxygenase inhibitors which have anti-inflammatory, analgesic and anti-pyretic effects and inhibit platelet aggregation [8]. Non-steroidal anti-inflammatory drugs are not subject to the stringent restrictions of narcotic drug laws, they do not depress respiration or impair gastro-intestinal motility and are not associated with dependence [8–10]. They are associated with histamine release and should be avoided in patients with a history of bronchospasm. They may also irritate the gastric mucosa with consequent indigestion or ulceration, and exacerbate renal and cardiac failure. However many studies have shown that with moderate doses the risk of these effects is small.

Ibuprofen is a NSAID that has a low incidence of serious side effects and is thought to be the safest NSAID available [11–13]. It is used extensively in the early management of soft tissue injuries in EDs throughout the world.

Although paracetamol is a safe and cheap analgesic, its analgesic effect is thought by many physicians to be inferior to that of the NSAIDs [14, 15]. A Cochrane clinical review has also commented in 2000 that there was ‘no good evidence that NSAIDs are more effective than paracetamol in acute musculoskeletal syndromes’ [16]. It was the most current information available at the time this study was planned.

Although there have been prospective studies comparing different oral NSAIDs in the treatment of post-operative pain [16–19], there have been limited large prospective, randomised controlled trials comparing ibuprofen and paracetamol head-to-head and in combination in the emergency department setting. In this study we aimed to compare the effect of paracetamol, ibuprofen and the combination of both in the treatment of soft tissue injury to compare their analgesic effects and any side effects experienced.

## Methods

### Trial design

This was a double-blind, double dummy, three-arm parallel, placebo controlled trial comparing oral paracetamol, ibuprofen and a combination of both, with an allocation ratio of 1:1:1. Ethical approval was obtained from the local institutional research ethics committee, the Joint Chinese University of Hong Kong—New Territories East Cluster Clinical Research Ethics Committee (reference no.: CRE-2004.266-T). The study was registered with ClinicalTrials.gov (no. NCT00528658). The study was planned in 2004 and 2005 and conducted between 2006 and 2009. However, competing work demands and priorities have led to long delays in the preparation of this manuscript which the principal investigator deeply regrets. We present it for publication now to complete the study and make our results publicly available.

### Inclusion criteria and exclusion criteria

This single centre study was conducted in the ED of a university teaching hospital serving the New Territories East region of Hong Kong. At the time of the study, the ED in Prince of Wales Hospital received over 170,000 new patients per annum and served a population of approximately 1,500,000.

All patients ≥18 years presenting to the ED with an isolated soft tissue injury without any suspicion of a clinically significant fracture between the hours of 9am to 5pm, Monday to Friday, were considered for the study.

Patients were excluded if they have contraindications to the use of paracetamol or ibuprofen including a history of indigestion, gastro-duodenal ulceration, bleeding disorders, recent anticoagulation, pregnancy, adverse reaction to paracetamol or NSAIDs/ibuprofen, cardiac failure, hepatic or kidney problems, rectal bleeding or chronic NSAID consumption, asthma, chronic obstructive airways disease, or chronic pain syndromes. Patients were also excluded if they had had analgesia in the four hours prior to recruitment, if they appeared to have other injuries or if they had a physical, visual or cognitive impairment that might make the use of the visual analogue scale unreliable.

### Randomisation, interventions and preparation of medication

Patients were randomly allocated to one of three treatment groups using a random number table [20, 21]. The random allocation sequence was generated by an independent researcher who had no role in patient recruitment, assignment of interventions, and outcome assessment.

Patients received either one true analgesic—paracetamol 1000 mg and placebo mimicking ibuprofen, or ibuprofen 400 mg and placebo mimicking paracetamol; or, two true analgesics—paracetamol 1000 mg and ibuprofen 400 mg. Paracetamol is given every 6 hours, and ibuprofen every 8 hours. The research nurse, the physician and the patient were blinded to the treatment allocations with the use of double placebo.

### Data collection: Pain score, observations and symptoms

Informed, written consent was obtained from each patient. Patients were given the study drugs and observed for a minimum of two hours in the ED. During this time patients were asked to record the level of pain using a visual analogue pain score (VAPS) [22] and initial adverse effects. Patients were also asked to self-record this information for three days after discharge during the morning, afternoon and at night. After discharge from hospital, patients were encouraged to return to the ED for further assessment in person. However, if the patient was not able to return for follow up, they were contacted by research staff via telephone. The adverse effects were collected by the research staff using a standardised data collection template.

The primary outcome of the study was the pain score at rest and pain score on activity in the first 2 hours and first 3 days following administration of oral paracetamol, oral ibuprofen or the combination of both. Previous research has also shown that ‘no worse than mild pain’ (30/100mm on a 100mm VAS), is an important outcome and cut-off for demonstrating an adequate response in analgesic trials [23], and therefore we have included the analysis in addition to the original protocol. Secondary outcomes included pain score at 28 days for those who required follow up by attending physicians, and the side effects experienced by patients.

Data was collected by a research nurse who was also responsible for ED staff information, motivation and feedback talks, for entry of data into a database, following up on the patients in the subsequent two days. Notes were made of re-attendance, protocol deviations, prescriptions of additional analgesia or other medication, and time to full recovery.

### Statistical analysis

Data was analysed on an intention to treat basis and all statistical analyses involved two-tailed tests. Baseline characteristics of the three treatments was analysed using the χ^2^ test or Mann-Whitney U test [20, 21]. The minimum clinically relevant reduction in pain score was considered to be 13mm [24, 25] and therefore differences of less than this value was considered clinically irrelevant. ANOVA was used to compare mean pain score reduction for the three groups for the primary outcome, chi-square test was used to compare the proportion of patients with adequate response and the adverse effects for the groups, and Kruskal-Wallis test to compare the overall satisfaction level. The standard deviation (SD) and 95% confidence interval (95% CI) was provided as appropriate. SPSS version 23 (IBM SPSS Statistics for Windows, Version 23.0. Armonk, NY: IBM Corp) was used for data analysis.

### Sample size calculation

In a previously published trial with two NSAIDs and paracetamol, adverse drug effects were seen in 1.4% in the least affected group and 4.4% in the worst affected group [26]. Using a two sided alpha of 0.05 and a beta of 0.2, the minimum numbers in each group to detect a difference in adverse events of that magnitude was 261 patients, which means 783 patients were required overall for the three groups.

## Results

**Fig 1:**
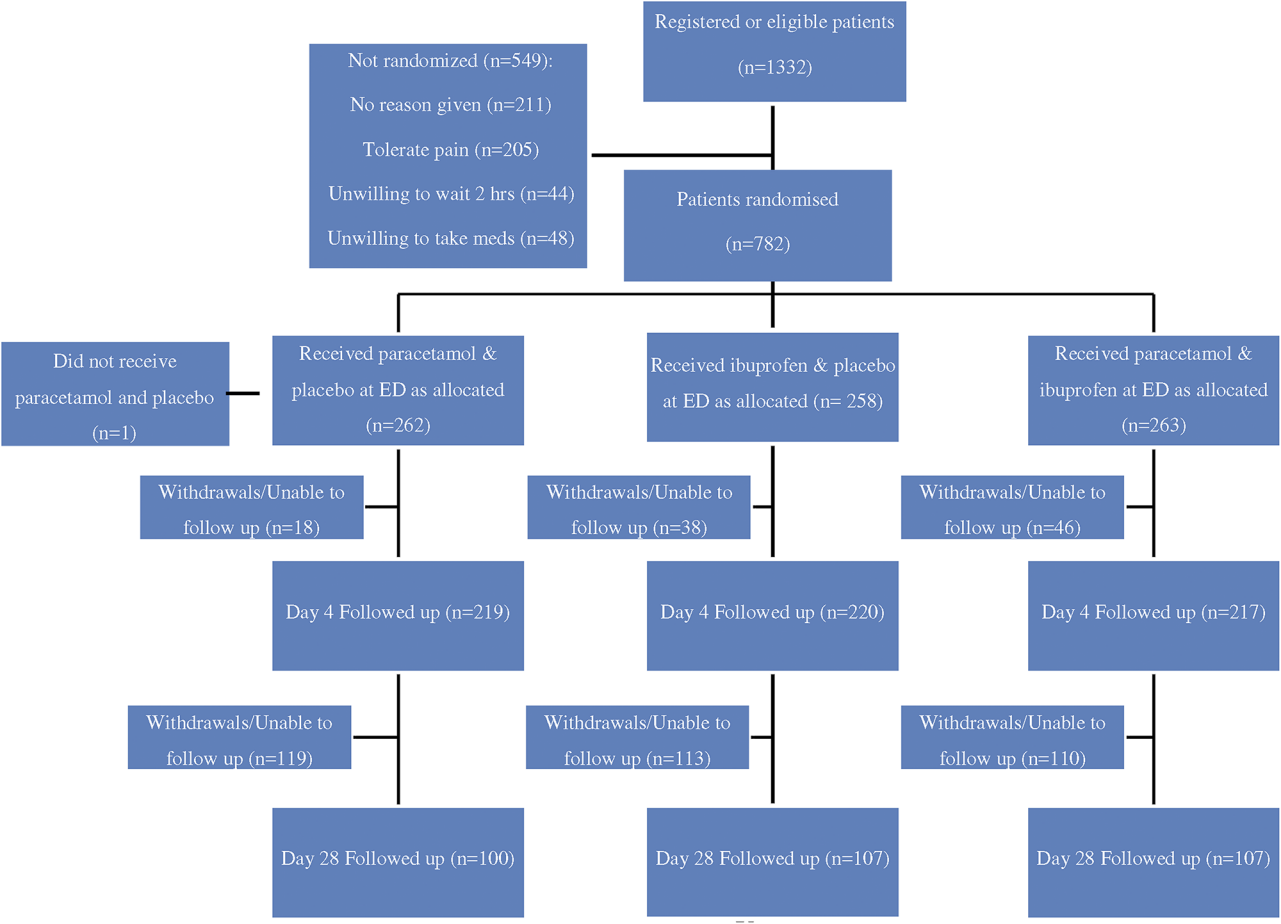
CONSORT flow chart describing progress of patients through randomised trial.

A total of 1332 patients who attended the ED with soft tissue injuries were screened, and 549 patients did not meet the inclusion and exclusion criteria. 783 were randomized into the three groups: 261 patients were randomised to paracetamol and placebo, 258 were allocated to receive ibuprofen and placebo and 263 were randomised to receive both. One patient in the paracetamol group was later excluded due to human error. 83.9% of patients completed follow up after 3 days, and 40.2% of patients completed follow up at 28 days. The CONSORT flow diagram is shown in Fig 1.

### Baseline characteristics and clinical outcomes

Baseline characteristics of the 782 participants are shown in Table 1. The three arms were similar in patient characteristics, types and sites of injury and baseline pain scores.

**Table 1:** Participants' characteristics (n = 782). Values are percentages of participants unless stated otherwise.

Variable	Paracetamol (n = 261)	Ibuprofen (n = 258)	Combined (n = 263)
Mean (SD) age (years)	38.9 (13.8)	39.0 (13.6)	39.1 (14.7)
No (%) of men	172 (66)	160 (62)	172 (65)
Median (interquartile range) time between injury and arrival at hospital (days)	0 (0–1)	1 (0–1)	0 (0–1)
**Types of injury**:			
Sprain	119 (46)	118 (46)	113 (43)
Contusion	89 (34)	84 (33)	87 (33)
Crush	17 (7)	20 (8)	17 (6)
Cut	36 (14)	36 (14)	46 (17)
**Site of injury**:			
Hand, Finger	70 (27)	54 (21)	66 (25)
Ankle	42 (16)	45 (17)	46 (17)
Back	38 (15)	45 (17)	48 (18)
Knee	29 (11)	21 (8)	22 (8)
Foot, Toe	21 (8)	38 (15)	22 (8)
Others	61 (23)	55 (21)	59 (22)
>1 injury site	19 (7)	26 (10)	30 (11)
Fracture	21 (8)	23 (9)	19 (7)
Wound	69 (26)	67 (26)	75 (29)
**Initial mean (SD) pain score**:			
At rest	30.5 (20.6)	30.9 (21.7)	32.9 (21.0)
With activity	63.1 (18.3)	61.7 (18.4)	60.5 (18.9)
Referred for orthopaedic assessment	32 (12)	26 (10)	19 (7)
Dressing	69 (26)	67 (26)	75 (29)

The mean age (SD) was 39 years (14.0), and 64.5% were male. Median time between injury and arrival at hospital was 1 day or less. The most common causes of injury included sprains (43–46%) or contusions (33–34%); 26–29% had a cut and less than 10% had a crush injury. The most common injury site was hands and fingers (21–27%), followed by the back (15–18%) and the ankle (16–17%). At rest, the mean (SD) pain scores at baseline for paracetamol, ibuprofen and combined treatment groups were 30.5 (20.6) mm, 30.9 (21.7) mm and 32.9 (21.0) mm respectively, and with activity were 63.1 (18.3) mm, 61.7 (18.4) mm and 60.5 (18.9) mm.

Before intervention, the percentage of those with VAS 30/100mm or above (moderate to severe pain) were similar for all three groups at rest (42.1–42.8%) and on activity (93.4–94.6%).

### Primary outcome—Change in VAS for pain at 2 hours and 3 days

#### VAS at rest and on activity at 2 hours

The reduction in mean pain score (95% CI) at rest in the initial two hours was 12mm (10–14 mm) in the paracetamol group, 12mm (10–15 mm)in the ibuprofen group, and 13mm (11–15 mm) in the combined group.

**Fig 2:**
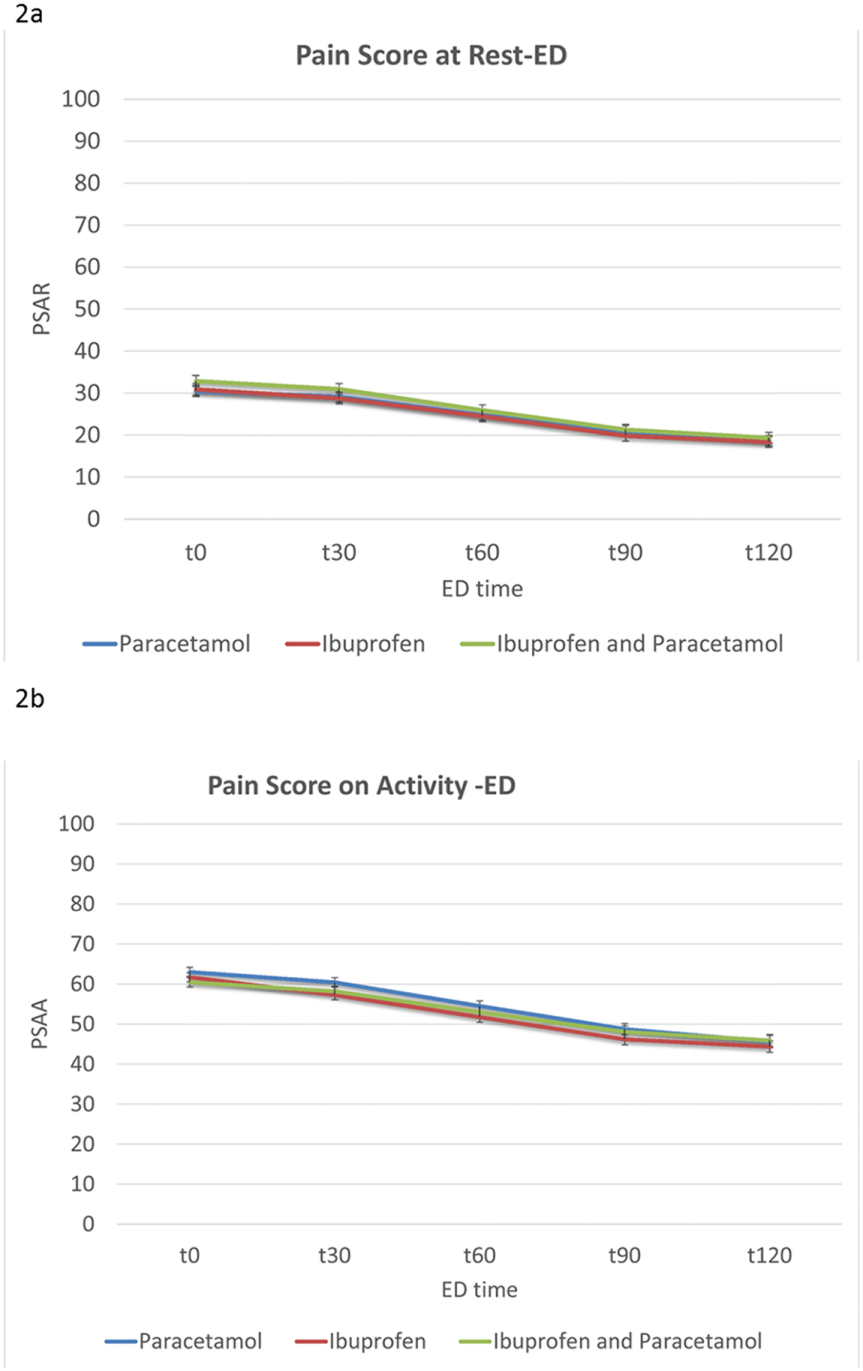
Reduction in VAS score (a) at rest and (b) on activity for the three treatment arms during the ED phase.

The reduction in mean pain score (95% CI) on activity was 17mm (15–19 mm) for paracetamol, 17mm (14–20 mm) for ibuprofen, and 15mm (12–17 mm) for the combined group. Changes in VAS for pain for the first two hours is shown in Fig 2.

All three treatment regimens were considered clinically effective in reducing pain at two hours. There were no significant differences in mean pain scores between the three intervention groups at rest (p = 0.68, ANOVA) or on activity (p = 0.22, ANOVA).

In patients with initial moderate to severe pain, the proportion of patients with adequate response (VAS <30mm) at 2 hours was not different for the three groups at rest (p = 0.98, chi-square) or on activity (p-0.83, chi-square).

#### VAS at rest and on activity at 3 days

The reduction in mean pain score (95% CI) at rest at 3 days was 18mm (15–21 mm) in the paracetamol group, 19mm (16–22 mm) in the ibuprofen group, and 20mm (17–23 mm) in the combined group.

**Fig 3:**
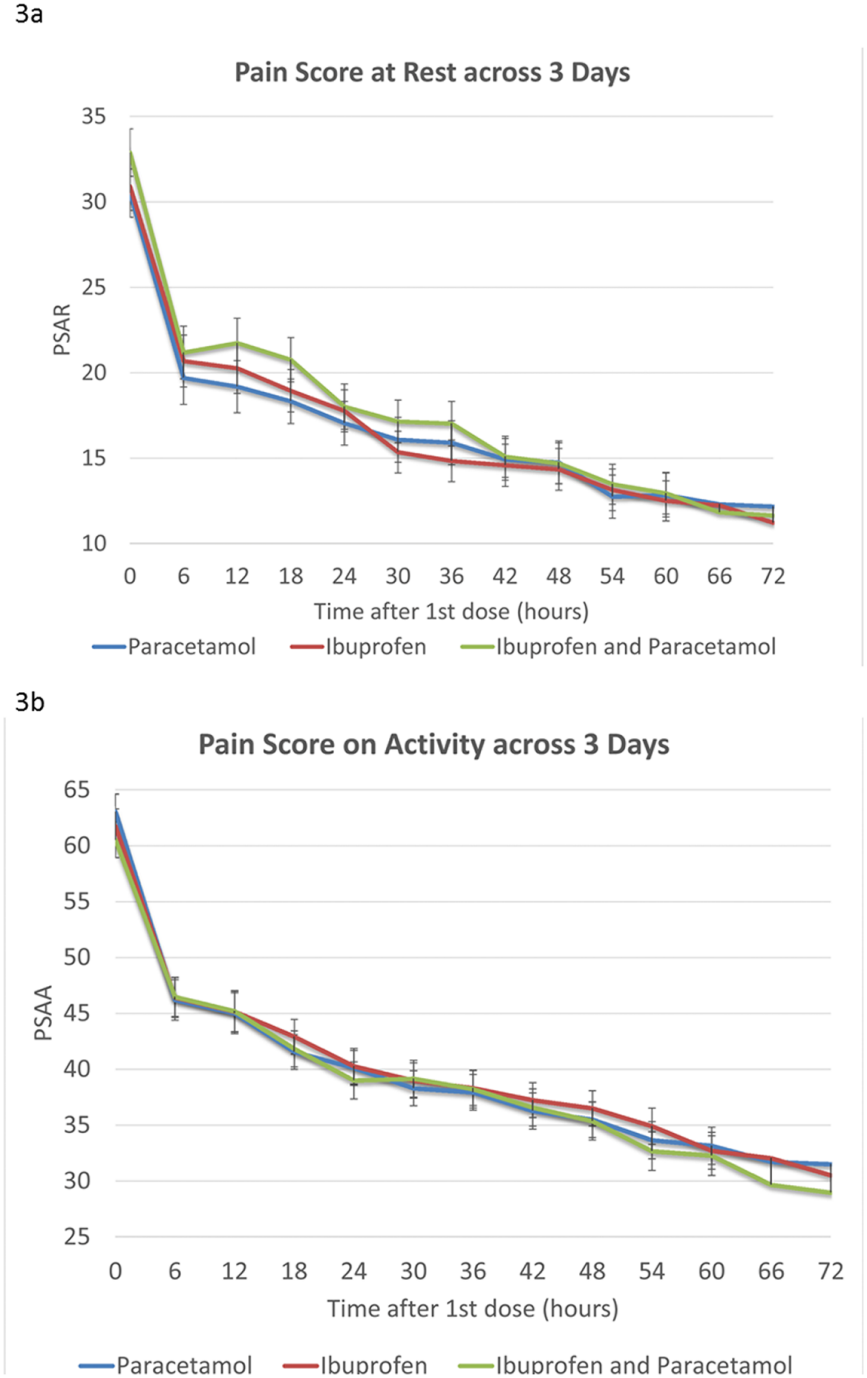
Reduction in VAS score (a) at rest and (b) on activity for the three treatment arms within 3 days.

The reduction in mean pain score (95% CI) on activity was 31mm (28–34 mm) for paracetamol, 30mm (26–34 mm) for ibuprofen, and 32mm (29–36 mm) for combined group. Change in VAS for pain for the first three days is shown in Fig 3.

Reduction in pain at rest and on activity for the three treatment groups were all clinically significant. Again the reductions were similar between the three intervention groups and were not statistically significant at rest (p = 0.86, ANOVA) or on activity (p = 0.57, ANOVA). The primary outcomes are summarised in Table 2.

**Table 2:** Summary of primary outcomes VAS and the univariate analysis.

Primary outcomes	Paracetamol (N = 261)	Ibuprofen (N = 258)	Combined (N = 263)	P value*
Reduction in VAS by 2 hours—at rest (95% CI)	12mm (10–14 mm)	12mm (10–15 mm)	13mm (11–15 mm)	0.68
Reduction in VAS by 2 hours—on activity (95% CI)	17mm (15–19 mm)	17mm (14–20 mm)	15mm (12–17 mm)	0.22
Reduction in VAS by 3 days—at rest (95% CI)	18mm (15–21 mm)	19mm (16–22 mm)	20mm (17–23 mm)	0.73
Reduction in VAS by 3 days—on activity (95% CI)	31mm (28–34 mm)	30mm (26–34 mm)	32mm (29–36 mm)	0.84

* The reduction in VAS were compared between groups using ANOVA

In patients with initial moderate to severe pain, the proportion of patients with adequate response (VAS <30mm) at 3 days was not different for the three groups at rest (p = 0.98, chi-square) or on activity (p-0.53, chi-square). The results were shown in Table 3.

**Table 3:** Proportion with ‘no worse than mild pain’ at baseline, 2 hours, 3 days and the proportion of adequate responders.

	Moderate to severe pain (≥30mm) initially	Adequate responders (reported mild pain) at 2 hours	P value*	Adequate responders (reported mild pain) at 3 days	P value*
Pain score at rest					
Paracetamol	103/243 (42.4%)	57/103 (55.3%)	0.980	59/74 (79.7%)	0.978
Ibuprofen	104/243 (42.8%)	59/104 (56.7%)		60/74 (81.1%)	
Ibuprofen and Paracetamol	102/242 (42.1%)	57/102 (55.9%)		58/72 (80.6%)	
Pain score on activity					
Paracetamol	226/242 (93.4%)	57/226 (25.2%)	0.829	77/152(50.7%)	0.531
Ibuprofen	227/243 (93.4%)	63/227 (27.8%)		87/169 (51.5%)	
Ibuprofen and Paracetamol	229/242 (94.6%)	61/229 (26.6%)		92/163 (56.4%)	

* The proportions of adequate responders were compared between groups using chi-square test

### Adverse events at end of ED phase and day 28

There was no serious adverse events which required admission, and no episodes of gastrointestinal haemorrhage, shortness of breath or chest pain were reported for all three arms. Minor adverse events were infrequent, with sleepiness the most frequently reported adverse event at the ED phase, and sleepiness, GI discomfort and dizziness at day 28. For each category of adverse events, there were no significant differences between the three treatment groups in the proportion of patients. The difference in the proportion of patients with more than one adverse event was also not significant (p = 0.7, chi-square). Table 4 shows the proportion of patients with adverse events in each treatment group at the end of ED phase and by day 28.

**Table 4:** Proportion of patients with adverse events in all randomized patients (N = 782).

	Intention to treat analysis*			
	Paracetamol (N = 261)	Ibuprofen (N = 258)	Combined (N = 263)	P value
**Extra analgesic given in ED**	26	31	33	
**Within the ED phase**				
Diarrhoea	0 (0%)	0 (0%)	0 (0.0%)	1.0
Dizziness	4 (1.5%)	1 (0.4%)	3 (1.1%)	0.4
GI discomfort	1 (0.4%)	3 (1.2%)	4 (1.5%)	0.4
Headache	0 (0%)	1 (0.4%)	3 (1.1%)	0.2
Nausea/ Vomit	1 (0.4%)	1 (0.4%)	1 (0.4%)	1.0
Rash	3 (1.1%)	3 (1.2%)	0 (0.0%)	0.2
Sleepiness	6 (2.3%)	12 (4.7%)	6 (2.3%)	0.2
Others	2 (0.8%)	0 (0%)	0 (0.0%)	0.1
**Within 28 days**				
Diarrhoea	2 (0.8%)	1 (0.4%)	1 (0.4%)	0.8
Dizziness	20 (7.7%)	14 (5.4%)	28 (10.6%)	0.1
GI discomfort	31 (11.9%)	41 (15.9%)	36 (13.7%)	0.4
Headache	13 (5.0%)	13 (5.0%)	18 (6.8%)	0.6
Nausea/ Vomit	11 (4.2%)	11 (4.3%)	10 (3.8%)	1.0
Rash	7 (2.7%)	11 (4.3%)	7 (2.7%)	0.5
Sleepiness	28 (10.7%)	22 (8.5%)	21 (8.0%)	0.5
Others	10 (3.8%)	11 (4.3%)	9 (3.4%)	0.9
Number of adverse events within 28 days				
0	183 (70.1%)	178 (69.0%)	180 (68.4%)	0.7
1	47 (16.9%)	51 (19.4%)	50 (19.0%)	
2	22(9.2%)	15 (6.2%)	22 (7.6%)	
3	6 (2.3%)	13 (3.9%)	8 (3.0%)	
>3	3 (1.5%)	1 (1.6%)	3 (1.9%)	

* The proportions of patients experiencing adverse events were compared between groups using chi-square test.

ED = Emergency Department

### Overall satisfaction level at day 28

Overall satisfaction level with the analgesic drug provided and with the clinical ED management was recorded on day 28. The overall satisfaction level was from 7–7.5/10. Again no significant difference was found between the three treatment groups with analgesic drug satisfaction (p = 0.3, Kruskal-Wallis test) and satisfaction with overall ED clinical management (p = 0.9, Kruskal-Wallis test). Table 5 shows the level of satisfaction for the analgesic effect of the treatment received.

**Table 5:** The level of satisfaction (maximum score 10) for the analgesic effect of the treatment received.

	Median (IQR)			P-value*
Treatment	Paracetamol (N = 215)	Ibuprofen (N = 216)	Combined (N = 219)	
Satisfaction with analgesia	7 (5–7.8)	7.5 (5–8)	7.5 (5–8)	0.3
Satisfaction with emergency department management	7.5 (5–8)	7.5 (5–8)	7.5 (5–8)	0.9

* The level of satisfaction with the analgesics and ED management were compared across groups using the Kruskal-Wallis test.

## Discussion

In this randomised controlled trial, there was no demonstrable difference in analgesic efficacy or side effects observed between paracetamol, ibuprofen or a combination of both in the ED treatment of soft tissue injuries. Although inflammation has been postulated to be the main reason for pain following soft tissue injury, this study has demonstrated that paracetamol is as effective as ibuprofen, a non-steroidal anti-inflammatory drug.

Others have recently reported the lack of efficacy of paracetamol as a single agent for the relief of low back pain [27]. While low back pain is typically non-traumatic, our study suggests that paracetamol may still have a role to play as a single agent analgesic in the initial treatment of soft tissue injuries. We did not examine pain scores or functional outcomes after 28 days, so it is not possible to directly compare our work with the available data on low back pain and paracetamol efficacy.

In a previous meta-analysis, ibuprofen was shown to be consistently superior to paracetamol in a range of conditions [28]. This was demonstrated particularly in acute post-operative pain, migraine, and osteoarthritis for chronic pain. With the use of fast acting formulations for ibuprofen, evidence suggested that they provide significantly better analgesia with more rapid initial reduction of pain intensity and reduced need for remedication [29, 30]. Further study is needed to prove the effectiveness of the fast acting formulations of ibuprofen in our study population.

Apart from the finding that paracetamol was as effective as ibuprofen, what was perhaps equally surprising was the lack of additional analgesic value with the combination of NSAID and paracetamol. Many authorities, including the WHO, have suggested the use of combination analgesia for some time now [31]. In a review of single does oral analgesics for acute postoperative pain in adults published in the Cochrane library, the number needed to treat (NNT) was clearly superior for the combination of ibuprofen and paracetamol compared to either drugs alone in achieving at least 50% maximum pain relief over 4–6 hours [32]. The NNT published in this 2015 review was 3.6 for paracetamol (975/1000mg), 2.5 for ibuprofen acid (400mg), 2.1 for ibuprofen fast acting (400mg) and 1.5 for ibuprofen 400mg + paracetamol 1000mg combination. It is not certain if the synergistic effects of adding a NSAID to paracetamol might be different in our study population, since a previous trial by Bondarsky et al also failed to demonstrate superiority for the drug combination for acute musculoskeletal injury [33]. It was acknowledged that the trial by Bondarsky et al could be underpowered and the negative results could have been contributed by the study design, but it has remained uncertain which patients might benefit from the ibuprofen and paracetamol combination in the ED.

Another surprise was the lack of serious adverse events noted in the study. There were no admissions with gastrointestinal bleeding, for example, which we had been specifically looking to identify given the propensity for Hong Kong Chinese to develop NSAID induced gastroduodenal ulceration and erosions [34]. Our study was powered to identify these adverse events and yet we did not identify any. We think it is unlikely that these patients were missed given the centralised nature of the local healthcare systems medical records, which are computerised, unless patients went to private medical facilities instead. We think this is unlikely given the suburban nature of the area that Prince of Wales Hospital serves.

As the population is aging all around the world and particularly in Hong Kong, we expect to see more and more elderly patients with co-morbidities attending the ED with soft tissue injuries. Although ibuprofen has a relatively safe side effect profile according to previous studies and our current findings, there will be increasing numbers of patients who have contraindications to the use of NSAIDs and the continuation of analgesic medication after discharge maybe a problem. The findings of this trial should reassure ED clinicians and others dealing with soft tissue injuries that providing an adequate dose of oral paracetamol is as effective as ibuprofen and the combination of both.

### Strengths and limitations

The strengths of the study lie in its randomised, controlled and double-blind design that enabled the analgesic efficacy and safety of paracetamol, ibuprofen and their combination to be explored in the ED management of soft tissue injury. The strength of the study lies in its pragmatic design and included a range of soft tissue injuries and body regions including ankle sprains and simple lower back pain. The trial was properly designed with an adequate sample size, and answers an important clinical question.

There are several limitations to the study. Firstly the baseline VAS at rest or on activity was not severe, as reflected by the percentage of those with ‘no worse than mild pain’ at baseline, and therefore the results may not apply to those with more severe injuries with higher baseline VAS scores. Secondly, only a modest dose of ibuprofen (1.2 gram daily) was used in this study, and therefore the potential analgesic effect of ibuprofen may be under-estimated. We acknowledge that the use of the acid base rather than the fast acting formulae of ibuprofen, and food intake with ibuprofen might have limited the analgesic effect. However this may also account for the lack of observed major adverse effects that has been described in the literature. It should be noted though that the typical dose of ibuprofen prescribed in Hong Kong is only 200mg three times daily, which is half the dose used in this study. The efficacy of such a small dose must be called into question given the results observed here.

The potential effect of ibuprofen might be further under-estimated as participants were asked to document their pain scores one hour after taking their analgesia four times daily while ibuprofen was administered eight hourly (three times daily).

## Conclusions

This trial has found that there is no difference in analgesic efficacy between oral paracetamol, oral ibuprofen and the combination of both paracetamol and ibuprofen for patients with mild to moderate pain after soft tissue injuries. Further studies including cost effectiveness data and the effects of gastroprotective agents in this setting may be of value in determining the overall place of ibuprofen and paracetamol in the management of soft tissue injury.

## Supporting information

S1 File CONSORT 2010 checklist CAG.doc. CONSORT Checklist.
(DOC)

S2 File Paracetamol and Ibuprofen protocol.doc. Trial research protocol.
(DOC)
