# Supplementary material for: Oral paracetamol and/or ibuprofen for treating pain after soft tissue injuries: Single centre double-blind, randomised controlled clinical trial
Source: PLoS One. 2018 Feb 6;13(2):e0192043. doi: 10.1371/journal.pone.0192043 (PMC5800651; doi:10.1371/journal.pone.0192043)
Supplement: S2 File — Trial research protocol. (DOC) [file pone.0192043.s002.doc]

Cost-effectiveness analysis of oral paracetamol and ibuprofen for treating pain after soft tissue limb injuries: double-blind, randomised controlled trial

**Principal Investigator**

Colin A Graham

Accident and Emergency Medicine Academic Unit, The Chinese University of Hong Kong

### **Introduction**

Non-steroidal anti-inflammatory drugs (NSAIDs), such as ibuprofen, and paracetamol are commonly used oral analgesics in emergency departments (ED) not only in Hong Kong but throughout the world. Every year, a great deal of money is spent prescribing analgesics for soft tissue injuries, and some of this is spent on expensive NSAIDs.

Studies have shown that there is little difference in analgesic effect among NSAIDs prescribed orally despite differences in cost although there may be differences in adverse effects. There are no large-scale (n>100), prospective, randomised studies comparing paracetamol with ibuprofen in the management of soft tissue injury.

Little is known about the relative efficacy of combinations of paracetamol and ibuprofen in the ED setting. As paracetamol is cheaper than most NSAIDs and may be as effective in the management of pain, possibly with fewer adverse effects, a large-scale, randomised, controlled trial is needed to answer questions of relative analgesic efficacy, safety and cost-effectiveness.

This has important implications for patients, physicians and health administrators within Hong Kong and, as these medications are used in many countries, will also have international relevance.

**Clinical Problem**

Soft tissue injuries (such as ankle sprains) commonly present to emergency departments (EDs), are painful and require adequate analgesia with either an oral non-steroidal anti-inflammatory drug (NSAID) and/or paracetamol [1]. The severity of injury is variable and may include simple ligament stretching, partial or complete ligament tears, cartilaginous damage or avulsion fractures. Most ankle sprains require early treatment such as rest, ice, compression, elevation and pain control, followed by active rehabilitation including ankle exercises and a graded increase in activity [2, 3].

Non-steroidal anti-inflammatory drugs are cyclo-oxygenase inhibitors which have anti-inflammatory, analgesic and anti-pyretic effects and inhibit thrombocyte aggregation [4]. Oral NSAIDs vary in price and degree of adverse effects but for comparable doses there is little difference in analgesic effect. Although there have been prospective studies comparing different oral NSAIDs in the treatment of post-operative pain [5-8], there have been no large prospective, randomised controlled cost-effectiveness trials comparing ibuprofen and paracetamol head-to-head and in combination.

Non-steroidal anti-inflammatory drugs are not subject to the strict restrictions of narcotic drug laws, do not depress respiration or impair gastro-intestinal motility and are not associated with dependence [4, 9, 10]. They are associated with histamine release and should be avoided in patients with a history of bronchospasm. They may also irritate the gastric mucosa with consequent indigestion or ulceration, and exacerbate renal and cardiac failure. However many studies have shown that with moderate doses the risk of these effects is very small.

Ibuprofen is an inexpensive NSAID which has a low incidence of serious side effects and is thought to be the safest NSAID available [11, 12, 13]. It is used extensively in the early management of soft tissue injuries in EDs throughout the world.

Paracetamol is a time-honoured, safe and cheap analgesic, although its analgesic effect is thought by many physicians to be inferior to that of the NSAIDs [14]. However, a Cochrane clinical review commented in 2000 that there was ‘no good evidence that NSAIDs are more effective than paracetamol in acute musculoskeletal syndromes’ [15].

# Economic Problem

NSAIDs and paracetamol are commonly prescribed analgesics both in hospital and the community yet may differ in efficacy, price and side effects. The respective prices of paracetamol (500mg) and ibuprofen (200mg) in Hong Kong are HK$ 0.1 and HK$ 0.1.

From a health services perspective, although these drugs are cheap (a so-called “little ticket item”), the quantities in which they are prescribed provide a considerable drain on the health care budget. We do not know the relative analgesic efficacy and adverse effects of NSAIDs compared with paracetamol and it may be that the latter would be a more cost-effective option in the management of soft tissue injuries. There is no evidence that ibuprofen is more effective than paracetamol in the management of soft tissue injuries. Paracetamol may confer the same analgesic effect as ibuprofen with fewer adverse events. A rare adverse effect of ibuprofen, in common with all NSAIDs, is upper gastrointestinal (GI) bleeding, which may cause a patient to be admitted for 2-3 days. At a cost of HK$3 000 per day for hospital costs, a few upper GI bleeds may change the cost-effectiveness profiles of the two drugs significantly.

The aim of this study is to perform a cost-effectiveness analysis comparing oral paracetamol, oral ibuprofen and a combination of paracetamol and ibuprofen in the management of painful soft tissue injury within an ED setting and after discharge. We hypothesise firstly that paracetamol 1g and ibuprofen 400mg administered orally for soft tissue injuries have equal analgesic efficacy; secondly that paracetamol has less adverse effects than ibuprofen; and finally that when all additional health care related costs are taken into account, that paracetamol will be the more cost-effective option.

Methods

This study will be conducted in the ED of the Prince of Wales Hospital (PWH), Shatin in collaboration with the Accident and Emergency Medicine Academic Unit of the Chinese University of Hong Kong (CUHK). The ED in PWH receives over 170 000 new patients per annum, serves a population of approximately 1 500 000, and admits 20% of those attending.

# Study Design

The proposal for this investigation is to conduct a prospective, double-blind, randomised controlled trial that will enable the analgesic efficacy, safety and cost-effectiveness of oral paracetamol and ibuprofen in the management of soft tissue injury to be established. A summary of the study design is presented in Appendix 1.

# Objectives

In the management of pain following isolated soft tissue injury, and within the context of an ED, we will:

1. establish and compare the analgesic efficacy of oral paracetamol, ibuprofen and the combination of the two drugs together;
2. establish and compare the safety (adverse effects) of oral paracetamol, ibuprofen and the combination of both;
3. perform a cost-effectiveness analysis comparing oral paracetamol, ibuprofen and the combination of both drugs together;
4. establish and compare patient satisfaction with oral paracetamol, ibuprofen and the combination of both drugs.

# Ethical approval

Ethical approval has been applied for from the local institutional Research Ethics Committee to conduct a pragmatic, prospective, randomised, double-blind, controlled study comparing oral paracetamol, ibuprofen and a combination of both in the management of soft tissue injury. Informed, written consent will be obtained from each patient.

The purpose and procedures of this study will be explained to each patient by a Cantonese speaking research nurse. An explanatory paragraph will accompany the written consent, which will be provided in both Chinese characters and in English (see Appendix 2). As part of the consent procedure patients will be informed that they may voluntarily withdraw from the study at any time. However the study will be performed on an intention to treat basis and all patients will be followed up for their subsequent management and outcomes.

# Sampling

Patients with isolated soft tissue injury will be considered to see whether they meet the appropriate inclusion criteria for the study (see Appendix 3). If they do so, then their consent will be sought and those that agree will be entered into the double-blind trial.

# Inclusion criteria and exclusion criteria

All patients ≥16 years presenting to the ED with isolated soft tissue injury without significant fracture between the hours of 9am to 5pm, Monday to Friday, will be considered for the study. The investigators do not consider it reasonable within the study budget to hire a research nurse for 24-hour or evening surveillance. All patients will be studied on an intention-to-treat basis.

Patients will be excluded if there is a history of peptic ulceration or haemorrhage, recent anticoagulation, pregnancy, adverse reaction to paracetamol or ibuprofen, renal or cardiac failure, hepatic problems, rectal bleeding, chronic NSAID consumption, asthma, chronic obstructive airways disease, chronic pain syndromes or prior treatment with analgesia for the same injury.

They will also be excluded if they have a physical, visual or cognitive impairment making use of the visual analogue scale unreliable (see Appendix 4).

# Randomisation, interventions and preparation of medication

Patients will be randomly allocated to one of three treatment groups using a random number table [16, 17]. Every patient will receive either:

1. one true analgesic – paracetamol – and one placebo mimicking ibuprofen
2. one true analgesic – ibuprofen – and one placebo mimicking paracetamol
3. two true analgesics – paracetamol and ibuprofen

Therefore one group will contain two analgesics – paracetamol and ibuprofen – thus allowing us to measure any combined effect and also to introduce a true double blind protocol.

Tablets are prepared as follows:

Tablet 1 – Paracetamol 500 mg [P],

Tablet 2 – Ibuprofen 200 mg [I],

Tablet 3 – Placebo for paracetamol [p]

Tablet 4 – Placebo for ibuprofen [i]

Therefore, each patient will be randomised to one of three groups:

- Group A will be prescribed 2x [P] four times a day, 2x [i] three times a day for 3 days.
- Group B will be prescribed 2x [p] four times a day, 2x [I] three times a day for 3 days.
- Group C will be prescribed 2x [P] four times a day, 2x [I] three times a day for 3 days.

Precoded envelopes with details of randomisation and group allocation will be opened by the research nurse. The medication will be prepared in prepacked packets outside of the Emergency Department and are therefore concealed from distributors. The research nurse will record the patient’s details and the medication to be administered in a Research Manual. A masterbase linking coded numbering and medication will be available in case the patient returns with adverse effects and the code needs to be broken.

# Data collection: pain score, observations and symptoms

During the study, patients will be managed as normal except for consent procedures. The patient will be given the study drugs, and observed over two hours for pain relief and initial adverse effects in the ED (see Appendix 4).

A 100mm, numbered, horizontal, visual analogue pain score (VAPS) [18] will be used for baseline measurements (t_0_), and at 20 (t_1_), 40 (t_2_), 60 (t_3_), 80 (t_4_), 100 (t_5_), 120 (t_6_) minutes after the first oral medication. Adverse events will be recorded and where possible analysed using the Morrow Index [19].

The patient will then be given a report card and visual analogue scale and asked to record the relevant information during the morning, afternoon and at night for three days after discharge (see Appendix 5). After discharge from hospital, the patient will either attend a follow up clinic or the research assistant will telephone the patient for follow up data. If patients have any concern regarding their condition they will be encouraged to return to the ED. Notes will be made of reattendance, deviations from protocol, prescription of additional analgesia or other medication, and time to full recovery.

Data will be collected by the research nurse (see appendix 4), who will also be responsible for ED staff information, motivation and feedback talks, for entry of data into a computer database and follow up on acceptability of the study and medication to the patient in the following two days. Data will be entered on to an Excel worksheet and subsequently analysed using SPSS v11.0.

# End Points

The study for each individual patient will end:

1. at a time specified by the patient if they wish to withdraw from the study (although every effort will be made to ensure that the patient has no untoward adverse effects),
2. from a senior attending A&E physician if he/she thinks advisable,
3. three days after the first dose of medication.

Having ‘left’ the study protocol, the patient may be given other analgesia of the attending physician’s choice. Every effort will be made to follow up such patients as they were entered into the study on an intention to treat basis.

# Cost

Professor Philip Jacobs of the University of Alberta, Canada, will provide expert support in developing the economic aspects of the study. Recent published guidelines will be used to establish the economic evaluation [20-27]. Since the patients presenting to the ED have varying severity of injury, it is to be expected that different patients would go through different treatment procedures before he/she is discharged. The randomised nature and power of the study should result in groups of equal demand except for those factors specifically associated with the three analgesics.

A list of specific activities relevant to the patient will be developed and we will capture all related services or activities including admission, readmission, reattendance, follow up at clinic and additional or altered analgesia regime. Unit costs (in HK dollars) will be estimated based on data obtained from the Finance Department at the Prince of Wales Hospital. These costs will include medical and nursing time as well as medical supplies, cost of drugs, diagnostic tests related to the injury, radiology, plaster cast or other limb support, and other drug treatments dealing with the side effects of the studying drugs.

A record will be made on a separate sheet of whether or not each of these factors have been utilised and of their frequency. Our separately developed costs will be applied to these activities in order to obtain a cost for each patient.

# Outcomes

The following outcomes will be sought:

1. analgesic efficacy both at rest and with movement,
2. presence, frequency and duration of adverse effects,
3. a cost-effectiveness analysis,
4. patient satisfaction with analgesia.

###### Cost measurements and data collection

Costs will be calculated according to activities, which included the preparation and administration of analgesics and other drugs, care of adverse events, hospitalisation related to adverse events.

# Cost effectiveness analysis

The primary efficiency measure is a cost-consequences analysis which is a comparison of costs with several different outcomes. Qualitative, rather than quantitative, descriptions will be made in comparing outcomes and efficiency measures. The sensitivity analysis of cost measures will be conducted with regard to observational periods.

# Statistical analysis

Data will be analysed on an intention to treat basis and all statistical analyses will involve two-tailed tests. As pain score and time data do not conform to the Gaussian distribution, non-parametric tests will be used to analyse data [28]. Baseline characteristics of the two treatments will be analysed using the χ^2^ test or Mann-Whitney U test [16, 17]. Time-to-event variables will be evaluated using the Kaplan-Meier product limit method, and the log-rank test used to compare the treatment groups. A regression line indicating the change in VAS pain score over time will be assessed and its slope used as a summary measure for each patient [29]. The median slope for each treatment group will be compared and analysed using the Mann-Whitney U test. The likelihood of achieving pain reduction will be compared and presented as hazard ratios. The minimum clinically relevant reduction in pain score is estimated as 13mm [30, 31] and therefore differences of less than this value will be considered clinically irrelevant.

**Sample size calculation**

In our previous smaller study with two NSAIDs and paracetamol, adverse drug effects were seen in 1.4% in the least affected group and 4.4% in the worst affected group. Using an alpha of 0.05 and a beta of 0.8, the minimum numbers in each group to detect that difference is 261 patients, which means we need 783 patients overall for the three groups. This will also sufficiently power the study for clinically relevant pain score differences (minimum clinically relevant difference is 13mm). We estimate that the difference in costs between the two groups may be as much as HK$50 per person from the healthcare perspective and the cost-effectiveness analysis will allow us to precisely quantify this.

Results of a pilot study [32]

A pilot study has been conducted in which 50 patients with painful soft tissue injuries were randomised and allocated into different analgesic groups: 16 patients received paracetamol and placebo (group 1); 12 patients received diclofenac (NSAID) and placebo (group 2); 11 patients received indomethacin (NSAID) and placebo (group 3); and 11 patients received paracetamol and diclofenac in combination (group 4).

**Baseline characteristics and clinical outcomes**

Baseline characteristics of these 50 patients with limb injuries in the four groups were similar. None were hospitalised, six need to have follow up in orthopaedic out-patient clinic for orthopaedic reasons and not because of adverse events to analgesia. Only one patient developed adverse effect to the analgesia. According to the doses and methods used in this study, there is no difference in pain relief both at rest and with activity two hours after the analgesic. No significant differences in pain relief in the first three days were observed.

**Cost analysis**

The three day courses of diclofenac, indomethacin and paracetamol are HK$9.00, HK$0.90 and HK$2.40 respectively. A three day course of ibuprofen would cost HK$1.80.

**Cost-effectiveness**

We noted there was no significant difference in pain reduction among the four groups despite different costs. Also, we did not identify any group having more side effects than the others, although the number of patients and adverse effects in the study was small. In summary, using diclofenac in relieving pain of soft tissue injuries seems to be not a cost-effective option. Paracetamol and indomethacin administration is the “dominant” strategy, with significantly lower costs, and comparable analgesic effect.

**Discussion**

This pilot study demonstrates that paracetamol and indomethacin may be less costly options than diclofenac in pain control for soft tissue injuries. It is also noted that the analgesic effect of paracetamol was no less than that of NSAIDs. This finding is potentially of great financial and clinical significance.

From a health service perspective, analgesic agents are being prescribed in large quantities which are a considerable drain on the health care budget. Therefore, an inexpensive, effective analgesic with fewer side effects should be welcomed by all physicians.

Although no patient develop side effects with NSAIDs in our study, this doesn’t mean that NSAIDs are free from side effects. There are two reasons to account for this. Firstly, the sample size in the pilot study may not be large enough to identify important adverse events. Secondly, we did not use high doses of NSAID in the study and side effects may therefore not be apparent. In addition, the high healthcare costs associated with admission for adverse events may greatly increase the cost and hence lower the cost-effectiveness for the causative drug. A single upper GI bleed caused by an NSAID may lead to a 2-3 day admission at HK$3 000 per day, which would have a significant impact on cost effectiveness overall from the healthcare perspective.

The strengths of the study lie in its randomised, controlled and double-blind design that enabled the analgesic efficacy, safety and cost-effectiveness of paracetamol, indomethacin and diclofenac to be explored in the management of soft tissue limb injury. The major criticism in this study is its small sample size which makes its validity questionable. Therefore, we need a large scale trial to verify these findings in the pilot study.

**Purpose and potential for implementation of results**

The management of pain remains one of the great challenges for ED’s worldwide and so rapid, effective and safe analgesic policies are essential for good patient care and patient satisfaction. The purpose of this study is to investigate whether oral paracetamol is at least as effective, and more cost-effective than ibuprofen in the management of acute pain following soft tissue injury in an ED. Doctors can use analgesic in a more cost-effective way, avoiding prescribing expensive analgesic without additional beneficial effects.

**Conflict of interest: None**

## References

1. Jantos TJ, Paris PM, Menegazzi JJ, Yealy DM. Analgesic practice for acute orthopaedic trauma pain in Costa Rican emergency departments. *Ann Emerg Med* 1996;**28**:145-50.
2. Wedmore IS, Charette J. Emergency department evaluation and treatment of ankle and foot injuries. *Emerg Med Clin North Am* 2000;**18**:85-113.
3. Rubin A, Sallis R. Evaluation and diagnosis of ankle injuries. *Am Fam Physician* 199;**54**:1609-18.
4. Ferreira SH. Prostaglandins, aspirin-like drugs, and analgesia. *Nature* 1975;**24**:200-203.
5. Collins SL, Moore RA, McQuay HJ, Wiffen PJ, Edwards JE. Single dose oral ibuprofen and diclofenac for postoperative pain. *Cochrane Database Syst Rev* 2000;(2):CD001548.
6. Romsing J, Ostergaard D, Drozdziewicz D, Schultz P, Ravn G. Diclofenac or acetaminophen for analgesia paediatric tonsillectomy outpatents. *Acta Anaesthesiol Scand* 2000;**44**:291-5.
7. Perez-Gutthan S, Garcia-Rodriguez LA, Duque-Oliart A, Varas-Lorenzo C. Low-dose diclofenac, naproxen, and ibuprofen cohort study. *Pharmacotherapy* 1999;**19**:854-9.
8. Davies NM, Skjodt NM. Choosing the right nonsteroidal anti-inflammatory drug for the right patient: a pharmacokinetic approach. *Clin Pharmacokinet* 2000;**38**:377-392.
9. British National Formulary. HMSO. London, 2004.
10. Benedetti C, Butler SH. Systemic analgesics. In Bonica JJ (Ed): *The Management of Pain*, ed 2. Philadelphia, Lee and Febiger, 1990:1640-1675.
11. Griffin MR, Piper JM, Daugherty JR, Snowden M, Ray WA. Nonsteroidal anti-inflammatory drug use and increased risk for peptic ulcer disease in elderly persons. *Ann Intern Med* 1991;**114**:257-63.
12. Garcia Rodriguez LA, Jick K. Risk of upper gastrointestinal bleeding and perforation associated with individual non-steroidal anti- inflammatory drugs. *Lancet* 1994;**343**:769-72.
13. Langman MJ, Weil J, Wainwright P, Lawson DH, Rawlins MD, Logan RF, Murphy M, Vessey MP, Colin-Jones DG. Risks of bleeding peptic ulcer associated with individual non-steroidal anti-inflammatory drugs. *Lancet* 1994;**343**:1075-8.
14. Owens PR. Prostaglandin synthetase inhibitors in the treatment of primary dysmenorrhea. *Obstet Gynecol* 1983;**61**:285-291.
15. Gotzsche PC. Extracts from “Clinical Evidence” Non-steroidal anti-inflammatory drugs. *BMJ* 2000;**320**:1058-61.
16. Kirkwood BR. Sampling methods. In: *Essentials of medical statistics.* Oxford: Blackwell Science, 1988; 167-172.
17. Bland M. *An introduction to medical statistics.* Oxford Publications. 1995. 2nd Edition.
18. Huskisson EC. Measurement of pain. *Lancet* 1974;**2**:1127-1131.
19. Morrow GR, Lindke J, Black P. Measurement of quality of life in patients: psychometric analyses of the Functional Living Index-Cancer (FLIC). *Qual Life Res* 1992;**5**:287-296.
20. Drummond MF, O’Brien B, Stoddart GL, Torrance GW. *Methods for the economic evaluation health care programmes.* Oxford: Oxford Medical Publications, 1997.
21. Bakker C, Hidding A, Linden S. Cost-effectiveness of group physical therapy compared to individualized therapy for ankylosing spondylitis: a randomized controlled trial. *J Rheum* 1994;**21**:264-268.
22. Marubini E, Valsecchi MG. *Analysing survival data from clinical trials and observational studies.* New York: John Wiley and Sons Inc. 1995
23. Byford S, Raftery J. Perspectives in economic evaluation. *BMJ* 1998;**316**:1529.
24. Palmer S, Byford S, Raftery J. Types of economic evaluation. *BMJ* 1999;**318**:1349.
25. Torgerson D, Raftery J. Main outcomes in economic evaluation. *BMJ* 1999;**318**:1413.
26. Williams RM. The costs of visits to emergency departments. *N Eng J Med* 1996;**334**:642-6.
27. Canadian Coordinating Office for Health Technology Assessment. *Guidelines for the economic evaluation of pharmaceuticals.* 2^nd^ edition. Ottawa. CCOHTA, 1997.
28. Gaddis GM, Salomone JA, Watson WA. Most linear analogue pain data should be analyzed by nonparametric statistical techniques (abstract). *Ann Emerg Med* 1992;**21**:629.
29. Matthews JNS, Altman DG, Campbell MJ, Royston P. Analysis of serial measurements in medical research. *BMJ* 1990;**300**:230-5.
30. Todd KH, Funk JP. The minimum clinically important difference in physician-assigned visual analog pain score. *Acad Emerg Med* 1996;**3**:142-6.
31. Todd KH, Funk KG, Funk JP, Bonacci R. Clinical significance of reported changes in pain severity. *Ann Emerg Med* 1996;**27**:485-9.
32. Man SY, Woo WK, Lam PKW, Rainer TH. Feasibility study comparing oral paracetamol and oral non-steroidal anti-inflammatory drugs for treating pain after musculoskeletal injury: a randomised, double blind, controlled trial. *Hong Kong J Emerg Med* 2004;**11**:78-84.

**APPENDIX 1 - STUDY DESIGN according to Consort Guidelines**

Registered or eligible patients (n=)

Randomisation

Not randomised (n=):

Patient choice (n=)

Disorder (n=)

Patients allocated to treatment (n=)

Received ibuprofen & placebo as allocated (n= )

Did not receive ibuprofen & placebo as allocated (n=)

Received paracetamol & placebo as allocated (n=)

Did not receive paracetamol & placebo as allocated (n=)

Followed up (n=)

Followed up (n=)

Withdrawals/Unable to follow up (n=)

Withdrawals/Unable to follow up (n=)

Received paracetamol & ibuprofen as allocated (n=)

Did not receive paracetamol & ibuprofen as allocated (n=)

Followed up (n=)

Withdrawals/Unable to follow up (n=)

**Fig 1 Flow chart describing progress of patients through randomised trial**

**APPENDIX 2 - CONSENT AND INFORMATION FORM**

**Trial of oral paracetamol and ibuprofen**

You have had a painful injury for which you would normally be offered pain killing tablets.

These drugs are chosen for their power to relieve your pain. All such medications have potential side effects such as nausea, vomiting, dizziness and allergic reaction. There have been extremely rare reports of kidney, respiratory and circulatory problems associated with these medications, but we obviously choose those that we know from all available information to be the safest and most effective. Despite our current knowledge we still need to learn more about their relative benefits and side effects so that we can treat our patients even better and more safely. For this we request the kindness of your help.

You can choose to take part in this search for better treatment whether you agree to have the pain relieving medications or not.

If you choose to have the medication you will be given one of three known successful pain relief combinations.

If you do not choose to have the medication you can still be very helpful in this search by giving the opportunity to compare what happens, after your injury, in the absence of analgesia.

Thank you for your involvement.

.............................................................................................................................................................

I agree to take part in this study. The details have been explained to me and I understand that I am free to leave the study at any time I wish.

.................................................................. ...............................................................

Signature of patient or guardian Signature of witness

.................................................

Date

PTO for Cantonese translation

**研究計劃同意書**

**囗服paracetamol和ibuprofen的試用**

閣下曾經因受傷而感到痛楚， 在正常情況下我們會給予閣下一些鎮痛藥。

我們選擇這些鎮痛藥是因為它們能夠舒緩閣下的痛楚。 這些藥物都有潛在的副作用例如噁心，嘔吐，頭暈及有敏感反應。 曾經有很少報告指出這些藥物會對腎臟， 呼吸及循環系統有壞影響， 但我們根據現有的所有資料而明顯地選擇這些被認為是最安全及最有效的藥物。 除了我們現有的知識之外， 我們需要知道這些藥物的益處和副作用， 這樣我們能夠更有效及更安全地治療我們的病人。 所以我們懇請閣下的幫忙。

無論閣下是否同意服用這些鎮痛藥物， 你可以選擇參與這項研究以取得更佳的治療。

如果閣下選擇接受這些藥物， 我們會將三種已知道是成功的鎮痛藥的其中一種給閣下試用。

如果閣下不選擇接受這些藥物， 閣下仍然可以對這項研究提供協助， 因閣下可以在受傷後而沒有止痛藥提供的情況下讓我們作一個比較。

我們感謝閣下的參與。

~~~~~~~~~~~~~~~~~~~~~~~~~~~~~~~~~~~~~~~~~~~~~~~~~~~~~~~~~~~~~~~~~~

**本人同意參與這項研究。 本人已獲得詳細的解釋並明白我可以隨時退出這項研究。**

病人或監護人簽名：__________________________

見證人簽名：__________________________

日期：_________________________

**APPENDIX 3 - INCLUSION AND EXCLUSION CHECKLIST**

***INCLUSION CRITERIA***

|  | Yes | No |
| --- | --- | --- |
| Is a limb fractured clinically? |  |  |
|  |  |  |

IF THE ANSWER IS NO THEN CONSIDER THEM FOR THE STUDY.

***EXCLUSION CRITERIA***

Does the patient answer YES to any of the following:

|  | Yes | No |
| --- | --- | --- |
| Have you any history of: |  |  |
| Indigestion |  |  |
| Gastro-duodenal ulcer |  |  |
| Bleeding disorders |  |  |
| Recent anticoagulation therapy (<3/12) |  |  |
| Pregnancy |  |  |
| Adverse reactions to NSAID’s/ibuprofen |  |  |
| Adverse reactions to paracetamol |  |  |
| Kidney problems |  |  |
| Heart failure |  |  |
| Liver problems |  |  |
| Rectal bleeding |  |  |
| Chronic NSAID consumption |  |  |
| Have you had any analgesia in the last 4 hours? |  |  |
| Is the patient aged less than 16 years? |  |  |
| Can the patient see the visual pain score? |  |  |
| Does the patient have a cognitive impairment? |  |  |
| Does the patient appear to have other injuries? |  |  |

**IF THE PATIENT ANSWERED “YES” TO ANY OF THESE EXCLUSION CRITERIA THEN EXCLUDE THEM FROM THE STUDY.**

**APPENDIX 4 - DATA FORM**

PATIENT DETAILS

Time of injury __ __:__ __

Extent of injury ______________________________________________________

______________________________________________________

Randomisation number __ __ __ __

Allocation group ____

|  | t_0_ | t_1_ | t_2_ | t_3_ | t_4_ | t_5_ | t_6_ |
| --- | --- | --- | --- | --- | --- | --- | --- |
|  | 0 mins | 20 mins | 40 mins | 60 mins | 80 mins | 100 mins | 120 mins |
|  |  |  |  |  |  |  |  |
| Intervention |  |  |  |  |  |  |  |
|  |  |  |  |  |  |  |  |
| Pain Score |  |  |  |  |  |  |  |
|  |  |  |  |  |  |  |  |
| Observations |  |  |  |  |  |  |  |
| Pulse |  |  |  |  |  |  |  |
| Systolic BP |  |  |  |  |  |  |  |
| Diastolic BP |  |  |  |  |  |  |  |
|  |  |  |  |  |  |  |  |
| Side effects |  |  |  |  |  |  |  |
| Headache |  |  |  |  |  |  |  |
| Dizziness |  |  |  |  |  |  |  |
| Nausea |  |  |  |  |  |  |  |
| Rashes |  |  |  |  |  |  |  |
| Indigestion |  |  |  |  |  |  |  |
| Vomiting |  |  |  |  |  |  |  |
| Bleeding |  |  |  |  |  |  |  |
| Other |  |  |  |  |  |  |  |
| Other |  |  |  |  |  |  |  |
|  |  |  |  |  |  |  |  |

**PAIN SCORE:**

**____________________________________________________**

###### No pain Worst pain

**0 1 2 3 4 5 6 7 8 9 10**

**APPENDIX 5 – TELEPHONE FOLLOW UP DATA FORM**

Name __ __ __ __ __ __ __ Randomisation number __ __ __Tel no. __ __ __ __ __ __ __ __

**PAIN SCORE:**

**____________________________________________________**

###### No pain Worst pain

**0 1 2 3 4 5 6 7 8 9 10**

|  | **Morning** | **Afternoon** | **Night** |
| --- | --- | --- | --- |
| **Time** | **11:00** | **17:00** | **23:00** |
|  |  |  |  |
| DAY 1 |  |  |  |
| Medication taken? | Y / N | Y / N | Y / N |
| Average pain score (at rest) |  |  |  |
| Average pain score (with activity) |  |  |  |
| Adverse events: |  |  |  |
| Headache |  |  |  |
| Dizziness |  |  |  |
| Nausea |  |  |  |
| Rashes |  |  |  |
| Indigestion |  |  |  |
| Vomiting |  |  |  |
| Bleeding |  |  |  |
|  |  |  |  |
| DAY 2 |  |  |  |
| Medication taken? | Y / N | Y / N | Y / N |
| Average pain score (at rest) |  |  |  |
| Average pain score (with activity) |  |  |  |
| Adverse events: |  |  |  |
| Headache |  |  |  |
| Dizziness |  |  |  |
| Nausea |  |  |  |
| Rashes |  |  |  |
| Indigestion |  |  |  |
| Vomiting |  |  |  |
| Bleeding |  |  |  |
|  |  |  |  |
| DAY 3 |  |  |  |
| Medication taken? | Y / N | Y / N | Y / N |
| Average pain score (at rest) |  |  |  |
| Average pain score (with activity) |  |  |  |
| Adverse events: |  |  |  |
| Headache |  |  |  |
| Dizziness |  |  |  |
| Nausea |  |  |  |
| Rashes |  |  |  |
| Indigestion |  |  |  |
| Vomiting |  |  |  |
| Bleeding |  |  |  |
|  |  |  |  |

**APPENDIX 6 - TIMETABLE OF WORK**

**Timetable of work**

| **Months** | **1** | **2** | **3** | **4** | **5** | **6** | **7** | **8** | **9** | **10** | **11** | **12** |
| --- | --- | --- | --- | --- | --- | --- | --- | --- | --- | --- | --- | --- |
| **Run in/pilot study** | * |  |  |  |  |  |  |  |  |  |  |  |
| **Study** |  | * | * | * | * | * | * | * | * |  |  |  |
| **Watershed** |  |  |  |  |  |  |  |  |  | * | * |  |
| **Itemised costing** |  |  |  |  |  |  |  |  |  |  | * |  |
| **Prepare report** |  |  |  |  |  |  |  |  |  |  | * | * |
| **Research Nurse** | * | * | * | * | * | * | * | * | * | * |  |  |
| **Visit of Health Economist** |  |  |  |  |  |  |  |  |  |  | * |  |
